# Increase of Blindness in the United States

**Published:** 1887-11

**Authors:** Lucien Howe

**Affiliations:** Buffalo


### Increase of Blindness in the United States.

BY DR. LUCIEN HOWE, OF BUFFALO.

(From paper read before the American Ophthalmological Society, July, 1887.)

Attention was called to the fact that, while the population during the ten years from 1870 to 1880 increased at the rate of 30 per cent., blindness during the same time increased over 140 per cent. By means of a diagram this percentage of increase in each State separately was shown. The statistics also show that blindness increased in an almost constant ratio from north to south in the United States, and that it decreased in the same way from east to west. This was also shown by colored maps. As for the causes, contagion was found to exercise the most important influence. Immigration was also considered as an important factor in view of the large number of contagious diseases of the eye introduced every year into the country, and the laxity or absence of quarantine regulations regarding them. As for the prevention, suggestions were offered, first, as to the care of new-born children; second, the isolation of suspicious cases in residential schools and other institutions, even for adults also; third, the instruction of the public as to the advisability of guarding against contagious forms of disease.

In coloring glass, the finest rubies, reds, purples, violets, etc., are produced by gold. The purple of Cassius, which is a mixture of the oxides of gold and tin, or some similar preparation of gold, is used. The coloring power of gold is so great that one part of gold will give a full rich body of color to from 600 to 1,000 parts of glass. The glass colored with gold can be made to assume a scarlet, carmine, rose, and ruby.

